# Carpenter’s Extract of Sarsaparilla

**Published:** 1835

**Authors:** 


### VII.—Carpenter's Compound Extract of Sarsaparilla.

This is a portable and elegant preparation of the ingredients (with some variation) of the celebrated Lisbon diet drink; and as such we beg leave to commend it to our country readers. It has been found, by such physicians of this city as have prescribed it, a valuable remedy in rheumatim, a variety of chronic diseases of the skin, old ulcers, subacute periostisis from mercury, and, with the addition of corosive sublimate, in secondary syphilis.

Mr. Carpenter is one of the most scientific pharmaceutic chemists of Philadelphia. His preparation may be had of all our respectable druggists.

